# Methicillin-Resistant *Staphylococcus aureus*^1^

**DOI:** 10.3201/eid1011.040797_10

**Published:** 2004-11

**Authors:** Fred C. Tenover, Michele L. Pearson

**Affiliations:** * Centers for Disease Control and Prevention, Atlanta, Georgia, USA

**Keywords:** MRSA, Methicillin-Resistant *Staphylococcus aureus,*

Methicillin-resistant *Staphylococcus aureus* (MRSA) is first and foremost a pathogen of healthcare settings. It is the most common pathogen associated with nosocomial infections in the United States, particularly nosocomial pneumonia and surgical site infections. It is also a frequent cause of bloodstream and skin and soft tissue infections. The percentage of *S. aureus* isolates resistant to oxacillin/methicillin in U.S. intensive care units increased from 30% to 40% in the mid-1990s to 57% in 2002.

Data from a recent Duke Infection Control Outreach Network survey indicate that of patients with healthcare-associated MRSA infections, 39% were from nursing homes, 37% had been hospitalized in the previous 90 days, 10% had received home health care, and 10% received dialysis. Data suggest that MRSA bacteremia is associated with an increased likelihood of death, longer hospital stays, and increased cost of hospitalization, when compared with bacteremia levels caused by methicillin-susceptible strains. Increasing resistance to vancomycin among MRSA also complicates therapy, which is already difficult because of multidrug resistance among healthcare-associated MRSA. Because spread of MRSA in healthcare settings is often clonal, hand hygiene and barrier precautions are often effective in interrupting spread. Targeted surveillance for MRSA is also a useful aid for infection control. Data from the Duke network indicate that the spread of MRSA can be curtailed in healthcare settings, given vigilance and adequate funding of infection control activities.

MRSA is now spreading in community settings. Reports from the early 1980s indicate that patients in the community without established risk factors for MRSA (i.e., recent hospitalization, residence in a long-term care facility, or dialysis) sought medical care with MRSA infections. In the late 1990s, four children in Minnesota and North Dakota died from community-associated MRSA infections. The isolates were susceptible to most non-β-lactam drugs, had pulsed-field gel electrophoresis (PFGE) profiles that differed from typical healthcare-associated MRSA, and contained the Panton-Valentine leukocidin toxin. Prospective surveillance for MRSA in Minnesota at 12 sentinel hospitals (6 in metropolitan areas and 6 in rural areas) indicated that community-associated MRSA patients were significantly younger than healthcare-associated MRSA patients and more likely to have skin and soft tissue infections than respiratory or urinary tract infections. A study in Texas showed that incision and drainage of abscesses due to community-associated MRSA was more effective management than administering antimicrobial agents alone, particularly since many patients were given ineffective antimicrobial agents (i.e., β-lactam agents).

Molecular analysis of the community-associated MRSA strains showed that the methicillin resistance gene *mecA* is typically carried on a much smaller genetic element than is seen in healthcare-associated MRSA. Four distinct elements, called staphylococcal chromosome cassette *mec* (or SCC*mec*), have been described. In the United States, SCCmec type II, which is approximately 60 kb in size and also carries an erythromycin resistance determinant, predominates among healthcare-associated MRSA, while SCCmec type IV, which is only ≈23 kb in length and carries no other resistance determinants, is typically associated with community-associated MRSA. Three major strain typing methods, PFGE, multi-locus sequence typing (MLST), and staphylococcal protein A typing (spa typing), are used to study the spread of MRSA. MLST identified a series of five major lineages (also called clonal complexes) of MRSA globally, while spa typing and PFGE subdivide this group into approximately a dozen epidemic clones. Virulence determinates for MRSA include a series of enterotoxins, toxic shock toxin, and

